# Blood pressure patterns in rural, semi-urban and urban children in the Ashanti region of Ghana, West Africa

**DOI:** 10.1186/1471-2458-5-114

**Published:** 2005-11-01

**Authors:** Charles Agyemang, William K Redekop, Ellis Owusu-Dabo, Marc A Bruijnzeels

**Affiliations:** 1 Institute of Health Policy and Management, Erasmus Medical Center, Rotterdam, The Netherlands; 2 Institute for Medical Technology Assessment, Erasmus Medical Center, Rotterdam, The Netherlands; 3 School of Medical Sciences, Kwame Nkrumah University of Ghana, Kumasi, Ghana

## Abstract

**Background:**

High blood pressure, once rare, is rapidly becoming a major public health burden in sub-Saharan/Africa. It is unclear whether this is reflected in children. The main purpose of this study was to assess blood pressure patterns among rural, semi-urban, and urban children and to determine the association of blood pressure with locality and body mass index (BMI) in this sub-Saharan Africa setting.

**Methods:**

We conducted a cross-sectional survey among school children aged 8–16 years in the Ashanti region of Ghana (West-Africa). There were 1277 children in the study (616 boys and 661 females). Of these 214 were from rural, 296 from semi-urban and 767 from urban settings.

**Results:**

Blood pressure increased with increasing age in rural, semi-urban and urban areas, and in both boys and girls. The rural boys had a lower systolic and diastolic blood pressure than semi-urban boys (104.7/62.3 vs. 109.2/66.5; p < 0.001) and lower systolic blood pressure than urban boys (104.7 vs. 107.6; p < 0.01). Girls had a higher blood pressure than boys (109.1/66.7 vs. 107.5/63.8; p < 0.01). With the exception of a lower diastolic blood pressure amongst rural girls, no differences were found between rural girls (107.4/64.4) and semi-urban girls (108.0/66.1) and urban girls (109.8/67.5). In multiple linear regression analysis, locality and BMI were independently associated with blood pressure in both boys and girls.

**Conclusion:**

These findings underscore the urgent need for public health measures to prevent increasing blood pressure and its sequelae from becoming another public health burden. More work on blood pressure in children in sub-Saharan African and other developing countries is needed to prevent high blood pressure from becoming a major burden in many of these countries.

## Background

High blood pressure has been identified as one of the leading causes of cardiovascular disease and premature mortality in the world [1]. In traditional African societies, high blood pressure, once rare, [2] is rapidly becoming a major public health burden [3-6]. The recent data show prevalence rates as high as 33% in some communities [3,4]. The increasing prevalence of hypertension is well reflected in the increasing stroke and cardiovascular disease morbidity and mortality [7-9].

In children, blood pressure tracking patterns confirm that persistent blood pressure elevation may be related to hypertension in adulthood [10,11]. The emerging data also suggest that primary hypertension is detectable and occurs commonly in the young [12]. In addition, the presence of elevated blood pressure in childhood has been linked with left ventricular hypertrophy [13]. As a result, in most western countries assessment and management of blood pressure in childhood is strongly recommended to promote improved cardiovascular health in adulthood [12]. Many epidemiological studies in various countries have been conducted to determine normal standard reference levels for age, sex, and body size [12,14]. However, in sub-Saharan African countries, blood pressure data on children and adolescents are very scarce. In Ghana for example, apart from the blood pressure profiles made in the 1970s on 5–12 year-olds in the Accra region, no other published data are available [14]. It is also unclear whether the recent rapid increases in blood pressure and prevalence of hypertension in adults [4,5,15] are reflected in children. Given the fast health transition towards non-communicable diseases and changes in lifestyle associated with urbanization [1,16], there is an urgent need for research on blood pressure in children so that appropriate cost-effective interventions can be introduced early in life to prevent the double burden of diseases in adulthood. The main purpose of this study was to assess blood pressure patterns among children in rural, semi-urban and urban settings in Ghanaian and to determine the association of blood pressure with locality and BMI in this Sub-Saharan African setting.

## Methods

### Study area

**Figure 1:**
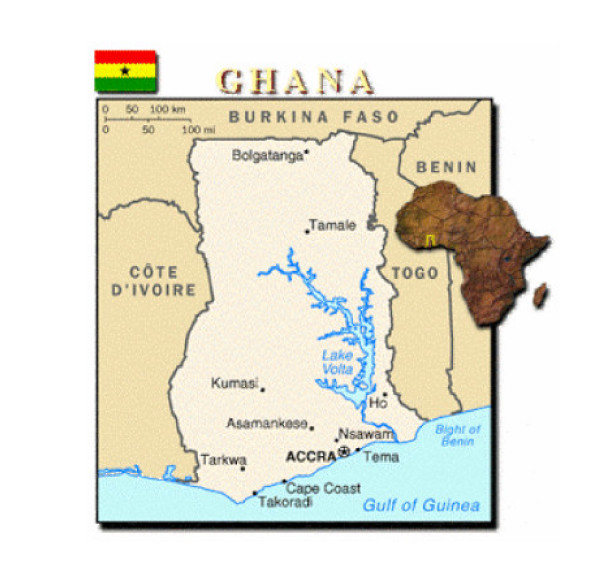
Map of Ghana.

Ghana is located on West Africa's Gulf of Guinea, only a few degrees north of the Equator with a total area of 238,540 square kilometres. It borders Côte d'Ivoire to the west, Burkina Faso to the north, Togo to the east and the Gulf of Guinea to the south (Figure 1). According to the 2000 census, the total population was about 18,800,000 with annual growth rate of 2.4%. The literacy rate is 74.8%. The predominant religion is Christianity (69%), followed by Islam (15.6%), traditional religions (8.5%), and other religions (6.9%). The life expectancy in 2001 was 56.2 years for men and 59.3 years for women. The GNP per capita in 2002 was US $1,900. Data for this study were collected in the Ashanti region, a region found near the centre of the country. It covers an area of 24,390 square kilometres representing 10.2% of the land area of Ghana. The region produces most of the country's cocoa, minerals and timber.

### Study design

Data were collected in seven primary schools among healthy children between the ages of 8 to 16 years in the cool season (August–September 2004). Four schools in the urban regional capital (Kumasi) were randomly selected from the schools' lists, two schools in semi-urban and one school in rural setting. Rural refers here to a village without electricity and main water supply, where the main occupation is subsistence farming. The sub-urban villages have electricity and main water supply, and the main occupation is subsistence farming. The schools were visited prior to the data collection in order to obtain permission from the relevant school principals as well as from the children. Following the local rules, the village chiefs and elders were also contacted in advance to obtain their permission. Because only physical measurements were made, only verbal informed consent was sought from the children and their guardians before measurements were taken. Data collection took place during normal school hours. In each school, all children age 8–16 years were included except for one big school in the regional capital where every other class was included. None of the children in the schools refused to participate in this study. Height was measured without shoes with a measuring tape to the nearest 0.5 cm. Weight was measured to the nearest 0.1 kg after removal of shoes, jackets, heavier clothing and pocket contents (using an Electronic Korona Profimed scale, Germany). Body mass index (BMI) was calculated as weight (kg) divided by height (m^2^). Children were classified as being overweight according to the BMI-for-age cut-off points corresponding to an adult BMI of 25 kg/m^2 ^[17]. Blood pressure and pulse were measured in the morning with a validated oscillometric automated digital blood pressure device (Omron M5-I monitor). Using appropriate cuff sizes, two readings with one-minute interval were taken on the right arm with the child in a seated position after at least five minutes rest. The mean of the two readings was used for analysis. The same trained staff made blood pressure measurements in all locations. In each school, prior to blood pressure measurements in children, all the teachers including the head-teacher had their blood pressure measured in front of the children to allay apprehension. The Committee on Human Research Publication and Ethics, Kwame Nkrumah University of Science and Technology, Kumasi, Ghana approved the study protocol.

### Data analysis

Age specific mean systolic and diastolic blood pressure levels were determined for rural, semi-urban and urban groups. The association between blood pressure and age was examined using linear regression analysis. Multiple linear regression analysis enabled age-adjusted comparisons of systolic and diastolic blood pressure levels to be made between gender and locality. Multiple linear regression analyses were also performed separately for boys and girls to assess the independent contribution of locality and BMI to systolic and diastolic blood pressure after adjustment for other factors associated with blood pressure in univariate analyses, including age and resting heart rate. All statistical analyses were performed using SPSS for Windows version 11.5 (SPSS Inc. Chicago, USA).

## Results

Table 1 shows the characteristics of the study population, anthropometrics, and blood pressure levels. There were 1277 participants in the study (616 boys and 661 females). Of these, 214 were from rural, 296 from semi-urban and 767 from urban settings.

**Table 1:** Characteristics of the population, anthropometrics and blood pressure levels by gender and locality

	**Sex**		**Boys**			**Girls**		
	**Boys (n = 616)**	**Girls (n = 661)**	**Rural (n = 111)**	**Semi-urban (n = 143)**	**Urban (n = 362)**	**Rural (n = 103)**	**Semi-urban (n = 153)**	**Urban (n = 405)**

Age (y)	12.9 (2.3)	13.0 (2.1)	12.7 (0.2)	11.8 (0.2)**	13.4 (0.2)***	12.6 (0.2)	12.5 (0.2)	13.2 (0.1)**
Height	147.2 (0.6)	148.8 (1.6)	141.5 (1.2)	137.8 (1.2)*	150.7 (0.7)***	142.2 (1.1)	142.0 (1.0)	149.5 (0.6)***
Weight	38.8 (0.4)	41.2 (0.4)***	34.8 (0.9)	32.1 (0.8)*	42.7 (0.5)***	36.1 (1.0)	36.4 (0.8)	44.3 (0.5)***
BMI	17.7 (0.1)	18.8 (0.1)***	17.1 (0.2)	16.5 (0.2)**	18.4 (0.2)***	17.6 (0.3)	17.7 (0.3)	19.5 (0.2)***
Overweight %	3.1	6.4***	0.0	1.4	4.6*	4.9	1.3	8.6
Pulse Rate	78.7 (0.5)	85.3 (0.5)***	82.5 (1.1)	78.3 (1.2)*	77.7 (0.7)***	88.9 (1.0)	79.5 (1.0)***	86.7 (0.7)
Systolic BP#	107.5 (0.4)	109.1 (0.4)**	104.7 (1.1)	109.2 (1.0)***	107.6 (0.6)**	107.4 (1.0)	108.0 (0.8)	109.8 (0.5)
Diastolic BP#	63.8 (0.4)	66.7 (0.3)***	62.3 (0.7)	66.5 (0.7)***	63.1 (0.4)	64.6 (0.9)	66.1 (0.7)	67.5 (0.5)**

Results are mean (SE); # Age adjusted. BMI = Body mass index; BP = Blood pressure; *P < 0.05, **P < 0.01, ***P < 0.001

### Blood pressure levels

**Figure 2:**
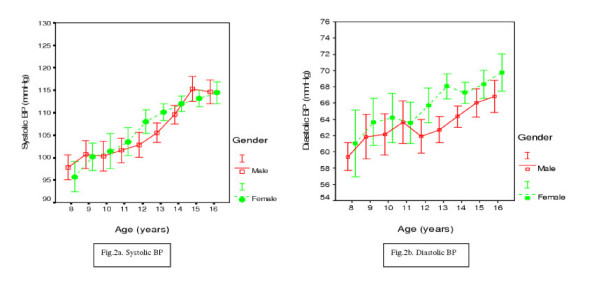
a and b: Systolic and diastolic blood pressure by age and gender (results are shown as mean and SE).

The systolic and diastolic blood pressure increased with increasing age in both boys and girls (figure 2a and 2b). This trend was also seen in rural, semi-urban and urban settings (Table 2). In a simple regression analysis, systolic and diastolic blood pressure increased by 3.0 mmHg and 1.0 mmHg per year respectively for rural children, and 2.0 mmHg and 0.6 mmHg per year respectively for semi-urban children, and 2.4 mmHg and 1.0 mmHg for urban children. All regression coefficients were statistically significant (p < 0.001). The age adjusted mean systolic and diastolic BP levels were significantly lower in boys than in girls.

**Table 2:** Mean (SE) Systolic and diastolic blood pressure (mm Hg) by age group and locality

		**Rural (n = 213)**			**Semi-urban (n = 296)**			**Urban (658)**	
Age (y)	n	Systolic BP	Diastolic BP	n	Systolic BP	Diastolic BP	n	Systolic BP	Diastolic BP

8	18	86.0 (2.4)	51.0 (1.7)	32	96.3 (1.5)	59.8 (1.4)	28	97.9 (1.6)	59.9 (1.4)
9	13	94.1 (2.7)	57.8 (1.8)	24	103.5 (1.5)	65.8 (1.5)	25	98.6 (1.4)	59.8 (1.8)
10	20	98.4 (2.3)	62.2 (1.7)	27	101.4 (2.1)	64.0 (1.5)	29	97.4 (2.3)	58.4 (2.1)
11	31	100.4 (1.9)	61.5 (1.5)	34	104.3 (1.6)	66.0 (1.6)	26	103.2 (1.9)	66.8 (1.6)
12	29	103.5 (2.6)	64.4 (1.9)	34	108.7 (2.1)	66.4 (1.5)	68	102.6 (1.1)	60.7 (0.9)
13	30	105.6 (1.7)	63.5 (1.4)	32	109.4 (1.7)	68.4 (1.2)	140	105.7 (1.0)	61.5 (1.0)
14	33	107.5 (2.0)	62.2 (1.0)	48	111.9 (1.8)	67.6 (1.2)	197	109.6 (1.2)	63.9 (0.6)
15	22	114.5 (2.6)	65.9 (1.7)	37	111.2 (1.8)	65.3 (1.4)	104	115.9 (1.6)	66.4 (1.1)
16	18	115.7 (2.3)	68.4 (1.9)	28	113.4 (1.7)	66.9 (1.5)	41	115.1 (1.4)	66.4 (1.2)
									
P value		<0.0001	<0.001		<0.0001	<0.0001		<0.0001	<0.0001

P value for linear regression

### Rural versus semi-urban and urban

As table 1 shows, the age adjusted mean systolic and diastolic blood pressures were significantly lower in rural boys than they were in semi-urban boys. Compared with urban boys, rural boys had a significantly lower age adjusted mean systolic blood pressure but a similar diastolic blood pressure. Among girls, the age adjusted mean systolic and diastolic blood pressure levels were lower in rural girls compared with urban girls, although only the diastolic blood pressure difference was statistically significant. No significant differences were found between rural girls and semi-urban girls except for a lower resting heart rate in semi-urban girls.

Table 3 shows that boys living in a rural area had lower systolic and diastolic blood pressure levels than other boys while BMI was positively associated with systolic and diastolic blood pressure. Among girls, rural locality was independently associated with lower diastolic blood pressure while BMI was positively associated with both systolic and diastolic blood pressure.

**Table 3:** Multiple regression analysis of factors associated with systolic and diastolic blood pressure

	**Systolic blood pressure**			**Diastolic blood pressure**		
**Boys**	Beta	SE	p-value	Beta	SE	p-value

Rural locality	-3.53	1.16	0.003	-1.71	0.84	0.043
BMI	1.63	0.24	<0.0001	0.60	0.17	<0.0001
Age	1.64	0.24	<0.0001	0.53	0.17	0.002
Heart rate	0.13	0.04	0.001	0.06	0.03	0.016
						
***R*^*2*^**	0.28			0.08		
						
**Girls**						
Rural locality	-0.07	1.09	0.325	-2.06	0.93	0.026
BMI	1.03	0.17	<0.0001	0.69	0.14	<0.0001
Age	1.68	0.22	<0.0001	0.54	0.19	0.005
Heart rate	0.16	0.03	<0.0001	0.13	0.03	<0.0001
						
***R*^*2*^**	0.25			0.12		

BMI = Body Mass Index

## Discussion

### Key findings

Blood pressure increased with age in rural, semi-urban and urban areas, and in both boys and girls. Blood pressure levels were lower in the rural population than in the semi-urban and urban populations. Locality and BMI were independently associated with blood pressure in both boys and girls.

### Discussion of key findings

The increase in blood pressure with age is consistent with previous reports in Ghana [4,5,14]. This was not only seen in semi-urban and urban settings but also in the rural setting. Less than half a century ago, ancestral African populations living traditional lives showed a lower mean blood pressure with little or no increase with increasing age, and low prevalence of hypertension [2]. However, in this present study, both systolic and diastolic blood pressure increased with increasing age (3 mmHg and 1.0 mmHg per year of age) in rural children. These findings seem to suggest that the protective effect against high blood pressure in rural settings in sub-Saharan Africa is fading. One possible explanation for these blood pressure trends in rural setting may be changes in lifestyles amongst these societies [18,19]. These findings do not bode well for the future, especially at a time when urbanization and westernization are proceeding at a faster rate [16]. In 2003 for example, stroke and cardiovascular disease were the 6th and the 7th most common causes of death in the Ashanti region of Ghana [8]. Increased blood pressure is a major contributing factor for stroke and cardiovascular diseases [7].

The lower blood pressure level found among rural school children is also consistent with the adult studies in Ghana [4,5] and other reports in sub-Saharan Africa [20,21]. For example, in Cappuccio et al's study, blood pressure was generally lower amongst rural dwellers than amongst semi-urban dwellers in Ghana [4]. In the present study, rural and urban differences in diastolic blood pressure still remained amongst both boys and girls even after adjustments for potential confounding factors. The reasons for these differences are unclear and further studies are needed to identify other factors that may contribute to the differences we observed. Boys had more favourable blood pressure profiles than girls. These gender differences in blood pressure patterns are consistent with earlier findings in the Greater Accra region of Ghana in both children [14] and adult [5] studies but contrast with findings of Cappuccio and colleagues in the Ashanti region of Ghana [4]. This is surprising given that Cappuccio et al's study was conducted in the same region as ours. Nonetheless, gender differences in blood pressure are generally inconsistent among African origin populations. In our recent report on blood pressure levels in ethnic minority children in the UK [22], blood pressure levels were generally more favorable for girls than for boys of African descent, while for adults, blood pressure patterns were more favourable for men than for women [23].

The strong and independent association between BMI and blood pressure is worrisome, especially for females in urban Ghana. Although the mechanisms by which BMI may lead to hypertension are poorly understood, it is now generally recognised that high BMI significantly increases the risk of hypertension. The impact of increasing BMI on high blood pressure has been clearly demonstrated in several populations [24,25]. Cooper and colleagues showed that the prevalence of high blood pressure increased as BMI increased across African descent populations [26]. In our study, both urban boys and girls had a higher BMI and were more likely to be overweight compared to their rural counterparts. Sinaiko and colleagues' prospective study showed that increases in weight and BMI in childhood were significantly associated with an increased risk of high blood pressure and other cardiovascular diseases in adulthood [27]. If the increasing frequency of overweight children is left unchecked while urbanization and westernization continue, it may lead to an increase in hypertension and other cardiovascular risks in future generations of Ghanaians [21]. In addition, the multiple regression models explained only 8% to 28% of the variance in systolic and diastolic blood pressures. This indicates that more work is needed to identify the other factors that contribute to an increase in blood pressure among children in this sub-Saharan African setting.

### Limitations

Like many population-based surveys, our blood pressure level was based on an average of two measurements at a single visit. A more precise estimate of blood pressure level would be obtained by multiple measurements obtained during several visits. Also, evidence suggests that during puberty blood pressure increases more rapidly, with a significant gender difference in the age of onset [28]. In the present study, pubertal status was not assessed and this may affect our study results. One other possible limitation is the use of blood pressure measurement techniques such as an automated oscillometric device, as opposed to auscultatory mercury manometers in children. However, the Omron M5-I device we used in this study has been shown to be a valid instrument for use amongst children [29]. Despite these potential shortcomings, the findings from this study are consistent with those from adult studies [4,5,15]. Our results may well be representative of the Ashanti Region of Ghana as a whole and may be used to formulate local health policy. Also, because this study provides a comprehensive assessment of children in three different localities, its results should serve as a wake-up call for sub-Saharan Africa countries and other developing countries to step up cost-effective measures early in life to prevent the double burden of diseases in adulthood.

## Conclusion and implications

This study shows an increase in blood pressure with age among these sub-Saharan African children in both rural and urban settings. This increase in blood pressure corresponds with the increasing prevalence of hypertension reported among adults. Blood pressure was positively associated with BMI in both boys and girls. These findings underscore the urgent need for public health measures to prevent high blood pressure and its sequelae from becoming another public health burden. In view of the scarcity of resources in Ghana as well as in many other developing nations, activities aimed at controlling increasing blood pressure in children have to compete with many other pressing health needs. Nevertheless, the long-term health problems that can result from increased blood pressure in children can be considerable. It is therefore important that measures be taken to reduce the risks of these problems and thereby optimize the health outcomes for future generations. Reducing the mean population blood pressure level by even as little as 2–3 mmHg could have a major impact in reducing associated morbidity and mortality [30]. A small pilot study of a nutritional education program in one village in Ghana resulted in the reduction in mean systolic and diastolic blood pressure by 6.4/4.5 mmHg within four weeks [31]. With careful implementation, such cost-effective measures may lead to an important reduction in blood pressure especially among city children, thereby sparing the next generation from high blood pressure related complications [31,32]. More work on blood pressure in children in sub-Saharan African and other developing countries is desperately needed, since high blood pressure is becoming a major public health issue in many countries.

## Competing interests

The author(s) declare that they have no competing interests.

## Authors' contributions

CA and MAB were responsible for study concept and design. CA and EO were responsible for data collection. CA, MAB and WKR were responsible for analysis and interpretation of data. CA drafted the manuscript and all were involved in critical revision of the manuscript. Statistical expertise was provided by WKR.

## Pre-publication history

The pre-publication history for this paper can be accessed here:


